# *CD79B* Y196 mutation is a potent predictive marker for favorable response to R‐MPV in primary central nervous system lymphoma

**DOI:** 10.1002/cam4.5512

**Published:** 2022-12-07

**Authors:** Junya Yamaguchi, Fumiharu Ohka, Chalise Lushun, Kazuya Motomura, Kosuke Aoki, Kazuhito Takeuchi, Yuichi Nagata, Satoshi Ito, Nobuhiko Mizutani, Masasuke Ohno, Noriyuki Suzaki, Syuntaro Takasu, Yukio Seki, Takahisa Kano, Kenichi Wakabayashi, Hirofumi Oyama, Shingo Kurahashi, Kuniaki Tanahashi, Masaki Hirano, Hiroyuki Shimizu, Yotaro Kitano, Sachi Maeda, Shintaro Yamazaki, Toshihiko Wakabayashi, Yutaka Kondo, Atsushi Natsume, Ryuta Saito

**Affiliations:** ^1^ Department of Neurosurgery Nagoya University Graduate School of Medicine Nagoya Japan; ^2^ Department of Neurosurgery Nagoya Central Hospital Nagoya Japan; ^3^ Department of Neurosurgery Konan Kosei Hospital Konan Japan; ^4^ Department of Neurosurgery Aichi Cancer Center Hospital Nagoya Japan; ^5^ Department of Neurosurgery Nagoya Medical Center Nagoya Japan; ^6^ Department of Neurosurgery Japanese Red Cross Aichi Medical Center Nagoya Daini Hospital Nagoya Japan; ^7^ Department of Neurosurgery Anjo Kosei Hospital Anjo Japan; ^8^ Department of Neurosurgery Toyohashi Municipal Hospital Toyohashi Japan; ^9^ Department of Hematology and Oncology Toyohashi Municipal Hospital Toyohashi Japan; ^10^ Department of Neurosurgery Nagoya Kyoritsu Hospital Nagoya Japan; ^11^ Division of Cancer Biology Nagoya University Graduate School of Medicine Nagoya Japan

**Keywords:** *CD79B*, *MYD88*, primary central nervous system lymphoma, rapid molecular diagnosis, R‐MPV

## Abstract

**Background:**

Rituximab, high‐dose methotrexate (HD‐MTX), procarbazine and vincristine (R‐MPV), has significantly prolonged the survival of patients with primary central nervous system lymphoma (PCNSL), but predictive factors for response to R‐MPV have not yet been investigated. Herein, we investigated the correlation of *MYD88* L265P and *CD79B* Y196 mutations, which are the most frequently found molecular alterations in PCNSL, with prognosis of patients with PCNSL treated with R‐MPV.

**Methods:**

We investigated the long‐term clinical course and status of *MYD88* and *CD79B* genes in 85 patients with PCNSL treated with R‐MPV or HD‐MTX treatment, and the correlation of these genetic mutations with prognosis.

**Results:**

R‐MPV achieved an excellent tumor control rate (61.6% and 69.9% of 5‐year progression‐free and overall survival rates, respectively). While *MYD88* L265P mutation had no significant effect on survival, patients with *CD79B* Y196 mutations exhibited prolonged survival (*p* < 0.05). However, the association of *CD79B* Y196 mutation with a better prognosis was not observed in the HD‐MTX cohort, which indicated that *CD79B* Y196 mutation was a predictive marker for a favorable response to R‐MPV. Furthermore, we established an all‐in‐one rapid genotyping system for these genetic mutations.

**Conclusions:**

In conclusion, *CD79B* Y196 mutation is a potent predictive marker for favorable response to R‐MPV in PCNSL. The rapid identification of *MYD88* L265P and *CD79B* Y196 mutations can be helpful not only for the accurate molecular diagnosis of PCNSL but also for the prediction of response to R‐MPV.

## Graphical abstract

CD79B Y196 mutation was a predictive marker for a favorable response to R‐MPV. We also established an all‐in‐one rapid genotyping system for this genetic mutation.

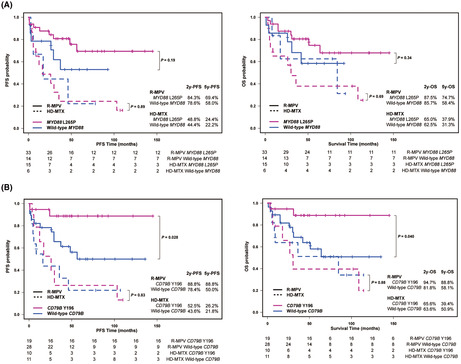

## 1 INTRODUCTION

Primary central nervous system lymphoma (PCNSL) is a form of malignant lymphoma that arises from the CNS, such as the brain parenchyma or eyes (ocular lymphoma). Although high‐dose methotrexate (HD‐MTX) regimen combined with conventional radiotherapy prolonged the overall survival (OS) of patients, neuropsychological evaluation revealed inferior cognitive function and quality of life (QoL), termed delayed neurotoxicity, after this combined therapy.^1^, ^2^ The rituximab, HD‐MTX, procarbazine and vincristine (R‐MPV) is one of the most promising induction regimens.^3^, ^4^ R‐MPV combined with reduced‐dose whole‐brain radiotherapy (rdWBRT; 23.4 Gy), with or without high‐dose cytarabine (HDAC), revealed favorable outcomes for patients newly diagnosed with PCNSL. Although R‐MPV has been introduced in many institutes, limited clinical studies have investigated long‐term efficacy and delayed neurotoxicity in patients with PCNSL treated with R‐MPV. Therefore, predictive factors for the response to R‐MPV have not yet been investigated.

In the last decade, molecular alterations in PCNSL have been uncovered. Activation of nuclear factor‐κβ (NF‐κβ) signaling is a hallmark of PCNSL.^5^ In particular, constitutive activation of *MYD88* and *CD79B* would initiate the signaling cascades of NF‐κβ; these are considered key mutations that promote lymphoma pathogenesis in immune‐privileged sites, because the frequencies of these mutations in PCNSL are higher than those in systemic malignant lymphoma.^6^, ^7^, ^8^, ^9^, ^10^, ^11^, ^12^, ^13^ Furthermore, of the gene mutations that occur in PCNSL, *MYD88* and *CD79B* mutations have hotspots and are simple to detect using Sanger sequencing or droplet digital PCR, which is advantageous for use in a daily clinical setting. However, the correlation of *MYD88* L265P and *CD79B* Y196 mutations with the prognosis of patients with PCNSL treated with R‐MPV has not been investigated in a large‐scale cohort subjected to a unified treatment regimen.

In this study, we retrospectively evaluated the long‐term clinical outcomes of 85 patients newly diagnosed with PCNSL and treated with R‐MPV or HD‐MTX, and further analyzed *MYD88* and *CD79B* status using the droplet digital PCR (ddPCR) method, a highly sensitive genotyping assay, to determine the correlation of *MYD88* L265P and *CD79B* mutations with the prognosis of patients with PCNSL treated with R‐MPV. Furthermore, we established a rapid genotyping system for *MYD88* and *CD79B* genes using an all‐in‐one genotyping system. This study revealed the clinical significance of *MYD88* and *CD79B* gene profiling on R‐MPV in PCNSL cases.

## 2 MATERIALS AND METHODS

### 2.1 Patient cohort

Sixty‐eight patients newly diagnosed with PCNSL, whose histological diagnosis was confirmed as diffuse large B‐cell lymphoma (DLBCL), were retrospectively enrolled. Forty‐seven patients received induction therapy with R‐MPV between 2009 and 2018 at the Konan Kosei Hospital and the Japanese Red Cross Aichi Medical Center Nagoya Daini Hospital, or between 2013 and 2018 at the Nagoya University Hospital. Twenty‐one patients received induction therapy with HD‐MTX with/without rituximab between 2009 and 2012 at the Nagoya University Hospital or between 2009 and 2018 at the Nagoya Medical Center. For validation, an additional 17 patients newly diagnosed with PCNSL, whose histological diagnosis was confirmed as DLBCL, were retrospectively enrolled. They received induction therapy with R‐MPV between 2007 and 2020 at the Anjo Kosei Hospital and Toyohashi Municipal Hospital. The age, sex, Karnofsky Performance Status (KPS) at diagnosis, Memorial Sloan‐Kettering Cancer Center recursive partitioning analysis (MSKCC RPA) score,^14^ location and number of lesions, and initial laboratory data of patients were reviewed from their medical records. Systemic lymphoma was excluded based on the results of fluorodeoxyglucose positron emission tomography or total body computed tomography and bone marrow examination before treatment.

### 2.2 Treatment regimen

Five 14‐day cycles of induction chemotherapy with R‐MPV were administered as follows: rituximab 375 mg/m^2^ on day 1, methotrexate 3.5 g/m^2^ on day 2, vincristine 1.4 mg/m^2^ on day 2, and procarbazine 100 mg/m^2^ on days 2–8 (only in odd cycles). In cases without a complete response (CR) after five cycles, two additional cycles of R‐MPV were performed. Following chemotherapy, patients who achieved CR received rdWBRT (23.4 Gy; 13 Fr). Patients who could not achieve CR after seven cycles or experienced tumor progression during induction therapy received conventional WBRT (40 Gy; 20 Fr). After completion of WBRT, two cycles of consolidation HDAC, at 3 g/m^2^ on days 1 and 2 of each cycle, were performed. Five 14‐day cycles of induction chemotherapy with HD‐MTX were administered as follows: methotrexate (3.5 g/m^2^) on day 1, with or without rituximab (375 mg/m^2^) on the day before methotrexate. Following five cycles of HD‐MTX, patients received WBRT (40 Gy; 20 Fr).

### 2.3 Evaluation of treatment response and toxicity

Responses were assessed by evaluating tumor sizes by contrast‐enhanced MRI according to the International Primary CNS Lymphoma Study Group. Individual responses were classified as CR, unconfirmed complete response (CRu), partial response (PR), stable disease (SD), or progressive disease (PD).^15^ MRI was performed after induction therapy, radiotherapy, and consolidation therapy. Adverse events (AEs) related to treatment were graded according to the Common Terminology Criteria for Adverse Event v5.0.

### 2.4 DNA extraction and ddPCR

Genomic DNA was extracted from unstained formalin‐fixed, paraffin‐embedded (FFPE) tissue using the QIAamp DNA FFPE Kit (Qiagen, Hilden, Germany). The concentration of the extracted genomic DNA was measured using a Qubit (Thermo Fisher Scientific, Waltham, MA, USA). Extracted genomic DNA (10–20 ng) was mixed with 10 μl of 2× ddPCR Supermix for probes (Bio‐Rad, Pleasanton, CA, USA) and 1 μl of 20× target (FAM) and wild‐type (HEX) probes. Details of each fluorescent probe sequence are described in Table S1. Each solution was mixed with 60 μl of droplet generation oil (Bio‐Rad), and droplets were generated using a QX200 Droplet Generator (Bio‐Rad). PCR was performed on a C1000 thermal cycler (Bio‐Rad) under the following thermal cycling conditions: 40 cycles of denaturation at 94°C for 30 s, annealing and extension at 53°C for *MYD88* L265P; 55°C for *CD79B* Y196H, Y196N, and Y196S; and 51°C for *CD79B* Y196D, Y196F, and Y196C for 60 s with a ramp rate of 2°C/s. The fluorescence intensity of each droplet was calculated using the QX200 Droplet Reader (Bio‐Rad) and analyzed using the Quanta Soft droplet reader software (Bio‐Rad).

### 2.5 Assessment of posttreatment QoL

Posttreatment QoL was assessed using the European Organization for Research and Treatment of Cancer (EORTC) Quality of Life Questionnaire C30 (QLQ‐C30, version 3.0) and brain cancer module (QLQ‐BN20).^16^ The QLQ‐C30 is composed of a global health status/QoL scale, functional scales (physical, role, emotional, cognitive, and social), and symptom scales/items (fatigue, nausea and vomiting, pain, dyspnea, insomnia, appetite loss, constipation, diarrhea, and financial difficulties). The QLQ‐BN20 is composed of supplementary scales/items (uncertainty about the future, visual disorder, motor dysfunction, communication deficit, headaches, seizures, drowsiness, hair loss, itchy skin, weakness of legs, and bladder control). All scales and single‐item measures ranged from 0 to 100. A high score indicates a high response level. Thus, a high score for a functional scale represents a high/healthy level of functioning, and a high score for the global health status/QoL represents a high QoL, but a high score for a symptom scale/item represents a high level of symptomatology/problems.

Patients who survived over the median follow‐up time answered the QLQ‐C30, version 3.0, and QLQ‐BN20 simultaneously at the time of the survey. Corrected scores were compared between patients who were irradiated with a dose of 23.4 Gy and those who were irradiated with a dose of over 23.4 Gy.

### 2.6 Genotyping of MYD88 L265P and CD79B Y196 mutations by i‐densy

The i‐densy is an all‐in‐one genotyping system that uses a fluorescent quenching probe (Q‐Probe) in which a fluorescent substance is bound to cytosine at the terminal end of the probe. The Q‐probe was designed to be quenched upon hybridization with the complementary strand. With increasing temperature, the duplex unravels at a melting temperature that depends on the strength of the bond between the Q‐probe and the tumor DNA at which point the fluorescence intensity recovers. Therefore, different fluorescent peaks are observed if there is a mismatched allele with the Q‐probe, which enables the detection of single‐nucleotide polymorphisms (SNPs). Q‐probes were designed to hybridize with the *MYD88* L265P mutant fragment and wild‐type *CD79B* fragment; the sequences of which are described in Table S1. Genomic DNA for genotyping by i‐densy was extracted from tumor samples by incubating tumor samples in 100 μl of distilled water at 95°C for 5 min. Genomic DNA (4 μl) and Q‐probes were applied to the cartridge. The i‐densy can identify four different targets simultaneously within 90 min.^17^, ^18^

### 2.7 Statistical analysis

OS and PFS rates were estimated using the Kaplan–Meier method, and the difference between the two groups was determined using the log‐rank test. Multivariate Cox regression analysis was performed using backward stepwise selection of variables based on the Akaike information criterion, and the statistical significance of the candidate variables, including clinical and genetic factors, was determined at *p* < 0.05, using log‐rank analyses. In multivariate analyses, multiple imputations of missing values were performed. Kaplan–Meier “survival” for Cox regression analysis and log‐rank test, “MASS” for stepwise Cox regression analysis, and “Amelia” for multiple imputations were performed using R software (version 3.5.1).

## 3 RESULTS

### 3.1 Comparison of clinical course of R‐MPV with that of HD‐MTX

**FIGURE 1:**
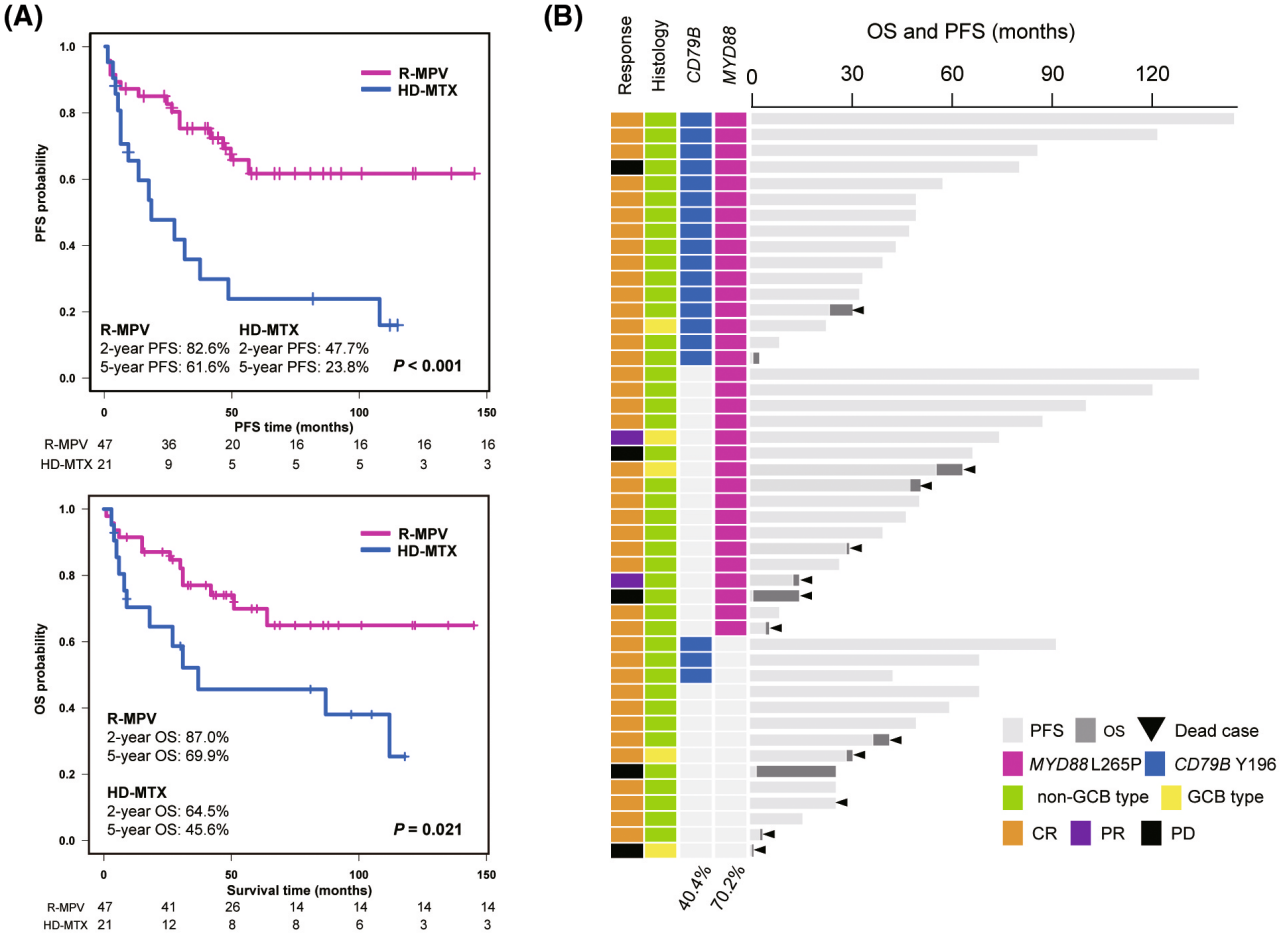
Summary of clinical course and molecular characteristics of the rituximab, high‐dose methotrexate (HD‐MTX), procarbazine and vincristine (R‐MPV) cohort. (A) Kaplan–Meier curve showing progression‐free survival (PFS: upper) and overall survival (OS: lower) of patients undergoing R‐MPV (pink line) and HD‐MTX (blue line) regimens. (B) Diagram showing initial response to R‐MPV (complete response [CR], partial response [PR], or progressive disease [PD]), histological molecular subgroup (germinal center B‐cell‐like [GCB] or non‐GCB type), *CD79B* Y196 and *MYD88* L265P status, and PFS (months) and OS (months) in each case. *MYD88* L265P and *CD79B* Y196 mutations were identified by droplet digital PCR (ddPCR).

The clinical characteristics of 68 newly diagnosed patients with PCNSL treated with R‐MPV combined with rdWBRT (*n* = 47) or HD‐MTX combined with conventional WBRT (*n* = 21) are described in Table 1. None of the patients had acquired immunodeficiency syndrome. The clinical characteristics of patients treated with R‐MPV were comparable to those of patients treated with HD‐MTX, except for sex. Among the 47 patients treated with R‐MPV, eight patients (17.0%) required additional R‐MPV cycles. Six of these eight patients achieved CR after additional R‐MPV cycles. The CR rate of the R‐MPV cohort (85.1%) was remarkably higher than that in the HD‐MTX cohort (47.6%). Mean follow‐up time was 50.1 and 43.7 months in the R‐MPV and HD‐MTX cohorts, respectively. The median PFS and OS of the R‐MPV cohort were not determined. The 2‐ and 5‐year PFS rates of the R‐MPV cohort were 82.6% and 61.6%, respectively, while those of the HD‐MTX cohort were 47.7% and 23.8%, respectively. The 2‐ and 5‐year OS rates of the R‐MPV cohort were 87.0% and 69.9%, respectively, while those of the HD‐MTX cohort were 64.5 and 45.6%, respectively. In particular, patients who achieved CR after R‐MPV showed extremely prolonged survival (2‐ and 5‐year PFS, 89.7% and 67.7%; 2‐ and 5‐year OS rates of 92.5% and 72.1%, respectively). The probabilities of PFS and OS were significantly higher in the R‐MPV cohort than in the HD‐MTX cohort (*p* < 0.001 and *p* = 0.021, respectively) (Figure 1A).

**TABLE 1:** Characteristics of patients in the high‐dose methotrexate (HD‐MTX) and rituximab, HD‐MTX, procarbazine and vincristine (R‐MPV) cohorts.

		R‐MPV (*n* = 47)	HD‐MTX (*n* = 21)	*p* value
Age, median (range)		66 (39–85)	64 (34–85)	0.84
Sex	Male	27 (57.4)	5 (23.8)	0.017
	Female	20 (42.6)	16 (76.2)	
KPS, median (%)		70 (20–100)	70 (50–100)	0.96
MSKCC RPA score (%)	Class1	3 (6.4)	2 (9.5)	0.78
	Class2	28 (59.6)	11 (52.4)	
	Class3	16 (34.0)	8 (38.1)	
Response (%)	CR	40 (85.1)	10 (47.6)	0.0035
	PR	2 (4.3)	4 (19.0)	
	SD	0 (0)	2 (9.5)	
	PD	5 (10.6)	5 (23.8)	
*MYD88* L265P mutation		33 (70.2)	15 (71.4)	1.0
*CD79B* Y196 mutation		19 (40.4)	10 (47.6)	0.61

Abbreviations: CR, complete response; HD‐MTX, high‐dose methotrexate; KPS, Karnofsky Performance Status; MSKCC RPA, Memorial Sloan‐Kettering Cancer Center recursive partitioning analysis; PD, progressive disease; PR, partial response; R‐MPV, rituximab, high‐dose methotrexate, procarbazine, and vincristine; SD, stable disease.

### 3.2 AEs and delayed neurotoxicity in R‐MPV‐treated patients

The incidence rates of severe hematological AEs, such as neutropenia and lymphopenia, were higher in the R‐MPV cohort (65.9% and 31.9%, respectively) than in the HD‐MTX cohort (4.8% and 9.6%; *p* < 0.001 and *p* = 0.12, respectively; Table S2).^19^ Although granulocyte‐colony stimulating factor was sometimes required to resolve severe neutropenia, in most cases, these AEs were improved without special treatment. However, lung infection, especially pneumocystis carinii pneumonia (PCP), can sometimes be critical. All three patients who developed PCP discontinued chemotherapy, and two of three patients died of PCP.

As delayed neurotoxicity develops a few years after WBRT and survivors over a longer period of time are more affected, we assessed the QoL of long survivors. Among the 68 patients, we identified 21 long survivors (12 patients who underwent R‐MPV with rdWBRT and nine who underwent HD‐MTX with conventional‐dose WBRT with survival over 50 months). These 21 patients answered questions of QLQ‐C30 and BN20 as indirect indicators of delayed neurotoxicity. No significant difference in mean survival time was noted between the rdWBRT and conventional‐dose WBRT groups (81 vs 103 months, *p* = 0.081) when they answered these questionnaires. Patients who underwent rdWBRT tended to have better scores on all scales and items compared to patients who underwent non‐reduced conventional‐dose WBRT. In particular, the patients who underwent rdWBRT revealed significantly superior pain scores in QLQ‐C30 and headache and communication deficits in BN20 compared to those in patients who underwent conventional‐dose WBRT (Figure S1).

### 3.3 Correlation of MYD88 or CD79B gene status and other clinical parameters with survival of patients treated with R‐MPV

Among 47 patients treated with R‐MPV, ddPCR analysis for *MYD88* L265P and *CD79B* Y196 mutations revealed 33 (70.2%) patients with *MYD88* L265P mutations and 19 (40.4%) patients with *CD79B* Y196 mutations (Figure 1B). Thirty‐six patients (76.6%) harbored either *MYD88* L265P or *CD79B* Y196 mutations. In detail, the 19 *CD79B* Y196 mutations included two (10.5%) Y196C, two (10.5%) Y196D, three (15.8%) Y196N, five (26.3%) Y196S, five (10.5%) Y196F, and five (26.3%) Y196H mutations. These data revealed that identifying *MYD88* L265P and *CD79B* Y196 mutations is quite effective for the diagnosis of PCNSL.

**FIGURE 2:**
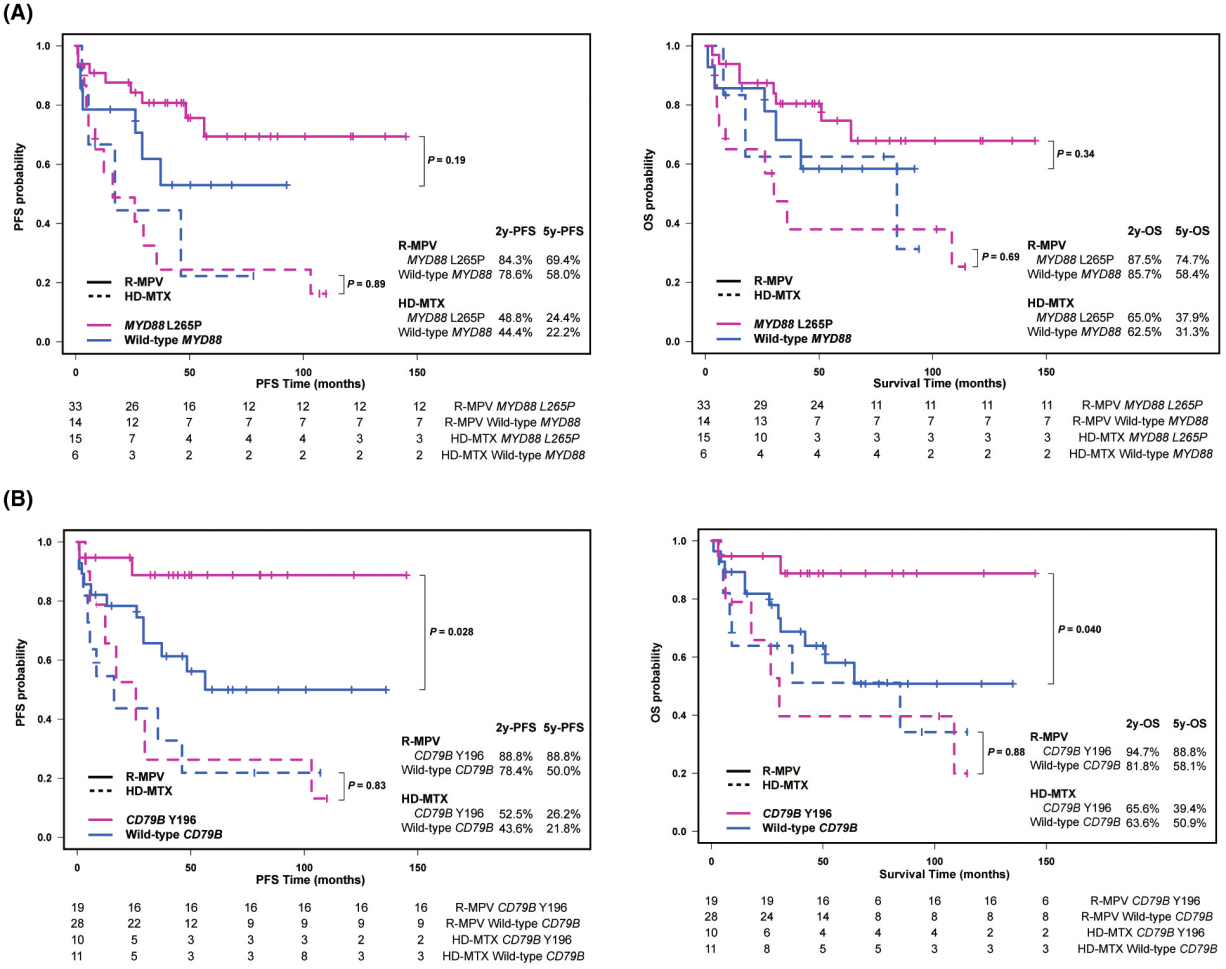
Correlation of *MYD88* L265P and *CD79B* Y196 mutations with survival. (A) Kaplan–Meier curve showing progression‐free survival (PFS; left) and overall survival (OS; right). The pink line shows survival of patients with *MYD88* L265P mutations and the blue line shows patients with wild‐type *MYD88*. The solid line indicates survival of patients treated with R‐MPV and the dotted line indicates patients treated with HD‐MTX. (B) Kaplan–Meier curve showing PFS (left) and OS (right). The pink line shows survival of patients with *CD79B* Y196 mutations and the blue line shows patients with wild‐type *CD79B*. The solid line indicates survival of patients treated with R‐MPV and the dotted line shows patients treated with HD‐MTX.

The impact of *MYD88* or *CD79B* status and other clinical parameters on the survival of patients treated with R‐MPV and HD‐MTX were tested using univariate analysis. *MYD88* L265P mutation did not significantly affect PFS and OS in patients treated with R‐MPV (*p* = 0.19 and *p* = 0.34, respectively), as this mutation did not affect those of patients treated with HD‐MTX (*p* = 0.89 and *p* = 0.69, respectively) (Figure 2A). Notably, *CD79B* Y196 mutations significantly affected PFS and OS in patients treated with R‐MPV (*p* = 0.028 and *p* = 0.040, respectively). However, this significant impact of *CD79B* Y196 mutation on prolonged survival was not observed in the HD‐MTX cohort, indicating that *CD79B* Y196 mutation is a predictive marker of R‐MPV (Figure 2B). Age was previously reported as a prognostic factor in PCNSL; however, age did not significantly affect PFS and OS in our R‐MPV cohort.^20^ Initial KPS (≤70) (*p* = 0.018 and 0.0054, respectively), multiple lesions (*p* = 0.0036 and 0.0016, respectively), deep lesions (*p* = 0.016 and 0.0076, respectively), and elevated serum soluble IL‐2 receptor (sIL‐2 R) (*p* = 0.0019 and 0.0012, respectively) significantly affected PFS and OS (Figure S2), which is consistent with previous reports. To determine the contributions of genetic and clinical factors to OS, we performed Cox proportional hazards regression modeling with backward stepwise selection of variables, incorporating the aforementioned factors, such as initial KPS (≤70), multiple lesions, deep lesions, elevated sIL‐2 R, and *CD79B* Y196 mutations. Elevated sIL‐2 R (hazard ratio [HR] = 6.65 [95% CI, 2.00*–*22.1], *p* = 0.0020) and *CD79B* Y196 mutation (HR = 0.18 [95% CI, 0.036*–*0.85], *p* = 0.031) were identified as independent predictors of survival (Table 2). These data suggest that *CD79B* Y196 mutation is an independent significant predictor of prolonged survival among patients with PCNSL treated with R‐MPV. To validate this finding, we examined the relationship between *CD79B* status and prognosis in 17 patients newly diagnosed with PCNSL and treated with R‐MPV. The clinical characteristics of the 17 patients are presented in Table S3. *CD79B* Y196 mutation also showed a clear trend toward better PFS and OS, although the difference was not statistically significant (PFS: HR = 0.54 [95% CI, 0.18*–*1.69], *p* = 0.228, OS: HR = 0.27 [95% CI, 0.08*–*0.95], *p* = 0.064) (Figure S3).

**TABLE 2:** Hazard ratio (HR) for overall survival (OS) in multivariate survival model.

Parameters	Hazard ratio (95% CI)	*p* value
Elevated sIL‐2 R	6.65 (2.00–22.1)	0.0020
*CD79B* Y196 mutation	0.18 (0.036–0.85)	0.031
KPS ≤70	0.32 (0.10–1.01)	0.053

### 3.4 Rapid genotyping of MYD88 L265P and CD79B Y196 mutations by i‐densy

**FIGURE 3:**
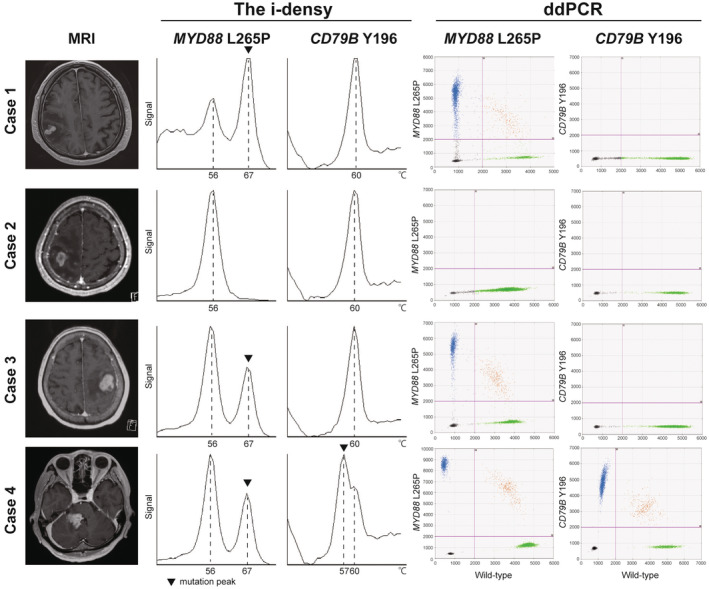
Genotyping of *MYD88* L265P and *CD79B* Y196 mutations using i‐densy and droplet digital PCR (ddPCR). Genotyping of *MYD88* L265P and *CD79B* Y196 mutations of four consecutive biopsies by i‐densy (left) and ddPCR (right). The fluorescence peak of *MYD88* L265P mutation was confirmed at 67°C and that of *CD79B* Y196 mutation was found at 57°C (black inverted triangle; left). The fluorescence peak of wild‐type *MYD88* or those of wild‐type *CD79B* were found at 56°C or 60°C, respectively. Two‐dimensional cluster (right) plot of droplet fluorescence of *MYD88* L265P and wild‐type *MYD88* dots and those of *CD79B* Y196 and wild‐type *CD79B* dots. FAM‐positive and HEX‐negative droplets (blue) include *MYD88* L265P or *CD79B* Y196. HEX‐positive and FAM‐negative droplets (green) include wild‐type *MYD88* or wild‐type *CD79B*. Cases 1 and 3 exhibited *MYD88* L265P mutation (+) and *CD79B* Y196 mutation (−). Case 2 exhibited *MYD88* L265P mutation (−) and *CD79B* Y196 mutation (−). Case 4 exhibited *MYD88* L265P mutation (+) and *CD79B* Y196 mutation (+).

We suggest that *CD79B* Y196 mutation is not only a reliable diagnostic marker combined with *MYD88* L265P mutation but also a predictive factor for response to R‐MPV. Therefore, we developed a rapid genotyping system for *MYD88* L265P and *CD79B* Y196 mutations in tissue samples using i‐densy. The fluorescence peak of *MYD88* L265P mutation was identified at 67°C, and that of *CD79B* Y196 mutation was identified at 57°C. The mutation peaks of *MYD88* L265P and *CD79B* Y196 mutations were detected in genomic DNA extracted from 1 mg of fresh‐frozen samples (Figure S4). We then applied this system to four consecutive biopsy samples from patients suspected to have PCNSL. In all cases, the status of *MYD88* and *CD79B* genes was obtained within 90 min by i‐densy. Further investigation revealed that these genotyping results were consistent with those obtained using ddPCR (Figure 3). The final pathological diagnosis of these cases was DLBCL.

## 4 DISCUSSION

Although PCNSL is one of the most devastating tumors of the CNS, recent developments in chemotherapy have significantly prolonged the survival of patients with PCNSL.^1^, ^3^, ^21^, ^22^ In this study, we investigated the long‐term clinical course of R‐MPV with rdWBRT for PCNSL using a large‐scale cohort. Although R‐MPV was related with higher frequencies of hematological AEs and PCP, R‐MPV achieved an excellent tumor control rate, and was considered a promising induction therapy for PCNSL. Recently, high‐dose chemotherapy (HDCT) with autologous stem cell implantation (ASCT) has been a promising consolidation therapy to minimize delayed neurotoxicity while maintaining equivalent tumor control compared to WBRT.^23^, ^24^ However, severe hematological AEs, technical difficulties, and long hospitalization periods prevent the widespread use of HDCT‐ASCT. WBRT is the most widely used consolidation therapy, and rdWBRT tends to reduce delayed neurotoxicity compared to conventional‐dose WBRT. Although the lack of a baseline score can be considered a limitation in this study, QoL was preserved in our cohort, as assessed from the QLQ‐C30 and BN20 scores. Therefore, our data supported that R‐MPV with rdWBRT is a promising and acceptable regimen for newly diagnosed PCNSL.

In systemic DLBCL, whose well‐established standard therapy is the R‐CHOP regimen, the predictive factors have been well documented.^25^ In contrast, the predictive molecular markers for response to R‐MPV for PCNSL have not been identified, although the landscape of molecular alterations in PCNSL has been unraveled. In this study, we investigated the clinical significance of *MYD88* and *CD79B*, common genetic variants in PCNSL, in the largest cohort with a unified treatment regimen (Table S4). We revealed that *MYD88* L265P mutation had no significant effect on the survival of R‐MPV‐treated patients with PCNSL consistent with the result of previous studies.^9^, ^26^, ^27^, ^28^, ^29^, ^30^, ^31^ In contrast, *CD79B* Y196 mutation was significantly correlated with prolonged survival in patients treated with R‐MPV. However, two previous studies showed that *CD79B* Y196 mutation had no significant effect on prognosis, and two other studies showed that *CD79B* mutation was significantly associated with inferior PFS or OS, which was in contrast to the results of this study.^9^, ^27^, ^29^, ^32^ In these studies, the sample size was small and administrated regimens were not unified. Additionally, in several studies, the treatment regimen was not described.^9^, ^27^, ^28^ These points could be the reason for the different results obtained in our study. On the other hand, our result was obtained from an analysis of as many as 47 cases treated with a single regimen, and Nayyar et al. showed that *CD79B* mutations significantly affected prolonged survival using 36 cases treated with just two regimens.^26^ Many of the patients in the other studies were administered a treatment regimen without rituximab. We hypothesized that *CD79B* Y196 mutation would affect the efficacy of rituximab, resulting in an improvement in the outcome of R‐MPV. In PCNSL harboring *CD79B* mutation, the activated PI3K‐mTOR axis acts in a pro‐survival manner.^33^ In the systemic DLBCL study, the activated PI3K‐mTOR axis was associated with poor prognosis in the CHOP‐treated group but not in the R‐CHOP‐treated group.^34^ Rituximab has been shown to synergize with mTOR inhibitors and suppress the PI3K‐mTOR axis alone.^34^ Rituximab may have been responsible for the tumor suppressive effect by not only the well‐known complement‐dependent cytotoxicity (CDC) and antibody‐dependent cell‐mediated cytotoxicity but also by PI3K‐mTOR axis suppression. Additionally, *CD79B* mutation increases surface BCR expression and induces chronic BCR signaling activation, which upregulates CD20 expression.^35^ Highly expressed CD20 appears to play an essential role in CDC induced by rituximab. Therefore, aberrantly expressed CD20 induced by *CD79B* mutation may have enhanced the effect of rituximab via CDC.^36^ The combination of rituximab with drugs that increase CD20 expression has been tested in several clinical trials.^37^, ^38^ Although, the clinical impact of the *CD79B* Y196 mutation is still controversial as described above, this study showed that *CD79B* Y196 mutation is a predictive marker of response to R‐MPV rather than a PCNSL prognostic marker. There is a clear trend that *CD79B* Y196 has an impact on better PFS and OS, and HRs consistent with those in the discovery cohort were obtained in the validation cohort (OS: HR = 0.18 [95% CI, 0.036*–*0.85] in discovery cohort vs HR = 0.27 [95% CI, 0.08*–*0.95] in the validation cohort). However, thelimitation of this study is that the validation cohort consisting of small number of cases did not reveal statistically significant differences in PFS and OS between the patients with *CD79B* Y196 and wild‐type *CD79B*. Further clinical studies with larger number of patients treated with R‐MPV are needed.

Bruton's tyrosine kinase (BTK) is a mediator of critical B‐cell signaling pathways, such as the B‐cell receptor (BCR) signaling pathway, involved in the pathogenesis of B‐cell malignancies. *MYD88* and *CD79B* mutations affect the BCR signaling pathway. Therefore, BTK inhibitors, such as ibrutinib and tirabrutinib, could be promising agents for patients with PCNSL, especially those harboring *MYD88* and/or *CD79B* mutations. Notably, a recent clinical study revealed that tirabrutinib showed a good response rate even in patients with PCNSL harboring wild‐type *MYD88* and/or *CD79B* genes.^39^ Additionally, clinical and fundamental studies of ibrutinib showed that *CD79B* mutation conversely attenuated the efficacy of ibrutinib by inducing alterations in the PI3K/mTOR pathway.^33^, ^40^ Although further investigation of the molecular mechanisms of BTK inhibitors is needed, the addition of BTK inhibitor to the R‐MPV might improve prognosis in patients with PCNSL harboring wild‐type *CD79B* because these patients were expected to be less responsive to the R‐MPV in our cohort.

The all‐in‐one genotyping system developed in this study enabled a simple, rapid, and accurate genotyping of *MYD88* L265P and *CD79B* Y196 mutations. Rapid genotyping of *MYD88* L265P and *CD79B* Y196 mutations is quite effective for intraoperative molecular diagnosis of PCNSL because it is sometimes difficult to distinguish PCNSL from inflammatory changes or other brain tumors. Furthermore, because CD79B mutation is related with response to R‐MPV or BTK inhibitor, rapid identification of CD79B status will be helpful in choosing postoperative treatment.

In conclusion, we demonstrated that the combination of R‐MPV with rdWBRT is associated with favorable long‐term survival in a large‐scale cohort and that *CD79B* Y196 mutation is associated with a good response to R‐MPV. The rapid and accurate genotyping system for *MYD88* L265P and *CD79B* Y196 mutations that we developed in this study will enable reliable rapid molecular diagnosis and early prediction of response to R‐MPV, which might help to determine the best treatment strategy for PCNSL.

## ETHICS STATEMENT

This study was performed in line with the principles of the Declaration of Helsinki. Approval was granted by the Institutional Review Board at Nagoya University Hospital (approval number: 2020–0207). Informed consent was obtained from all individual participants included in the study. Registry and the Registration No. of the study/trial was N/A. Animal Studies were N/A.

## AUTHOR CONTRIBUTIONS

**Junya Yamaguchi:** Conceptualization (equal); data curation (lead); formal analysis (equal); investigation (lead); methodology (equal); project administration (equal); writing – original draft (equal); writing – review and editing (equal). **Fumiharu Ohka:** Conceptualization (lead); funding acquisition (lead); investigation (equal); project administration (lead); writing – original draft (lead); writing – review and editing (lead). **Chalise Lushun:** Data curation (equal); investigation (equal); project administration (equal); writing – original draft (equal). **Kazuya Motomura:** Data curation (equal). **Kosuke Aoki:** Data curation (equal); formal analysis (equal); methodology (equal). **Kazuhito Takeuchi:** Data curation (equal). **Yuichi Nagata:** Data curation (equal). **Satoshi Ito:** Data curation (equal). **Nobuhiko Mizutani:** Data curation (equal). **Masasuke Ohno:** Data curation (equal). **Noriyuki Suzaki:** Data curation (equal). **Syuntaro Takasu:** Data curation (equal). **Yukio Seki:** Data curation (equal). **Takahisa Kano:** Resources (equal). **Kenichi Wakabayashi:** Resources (equal). **Hirofumi Oyama:** Resources (equal). **Shingo Kurahashi:** Resources (equal). **Kuniaki Tanahashi:** Data curation (equal). **Masaki Hirano:** Data curation (equal). **Hiroyuki Shimizu:** Data curation (equal). **Yotaro Kitano:** Data curation (equal). **Sachi Maeda:** Data curation (equal). **Shintaro Yamazaki:** Data curation (equal). **Toshihiko Wakabayashi:** Data curation (equal). **Yutaka Kondo:** Data curation (equal). **Atsushi Natsume:** Data curation (equal). **Ryuta Saito:** Conceptualization (equal); data curation (equal); project administration (equal); writing – original draft (equal); writing – review and editing (equal).

## FUNDING INFORMATION

This study was performed as part of the research grant of THE HORI SCIENCE AND ARTS FOUNDATION (F. Ohka).

## Supporting information


Figure S1.

Figure S2.

Figure S3.

Figure S4.

Table S1.

Table S2.

Table S3.

Table S4.

## Data Availability

Data sharing is not applicable to this article as no new data were created or analyzed in this study.
