# Supplementary figures and images for: CD79B Y196 mutation is a potent predictive marker for favorable response to R‐MPV in primary central nervous system lymphoma
Source: Cancer Med. 2022 Dec 7;12(6):7116–26. doi: 10.1002/cam4.5512 (PMC10067082; doi:10.1002/cam4.5512)

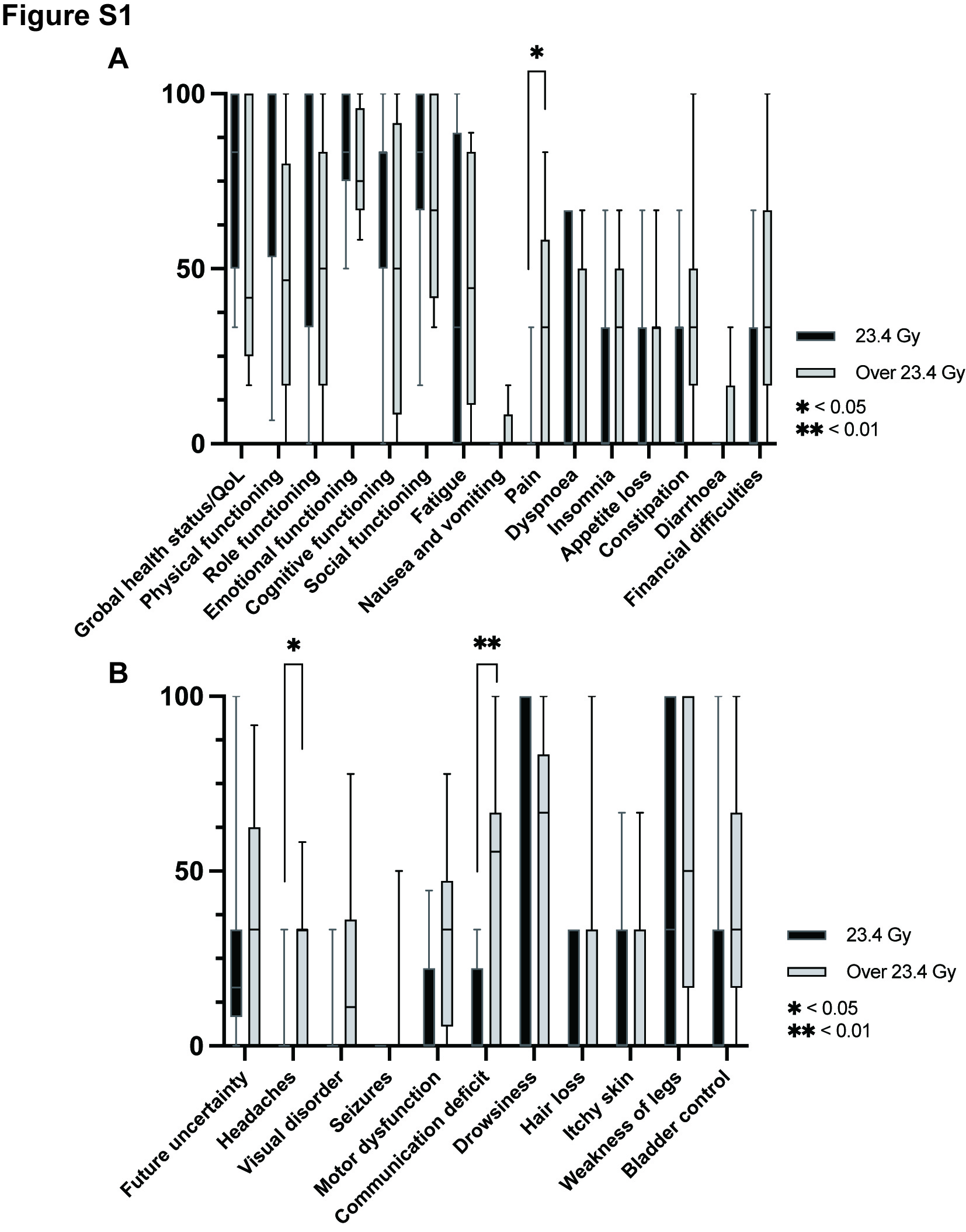

Supplement: Supplementary file 1 — Figure S1. [file CAM4-12-7116-s003.jpg]

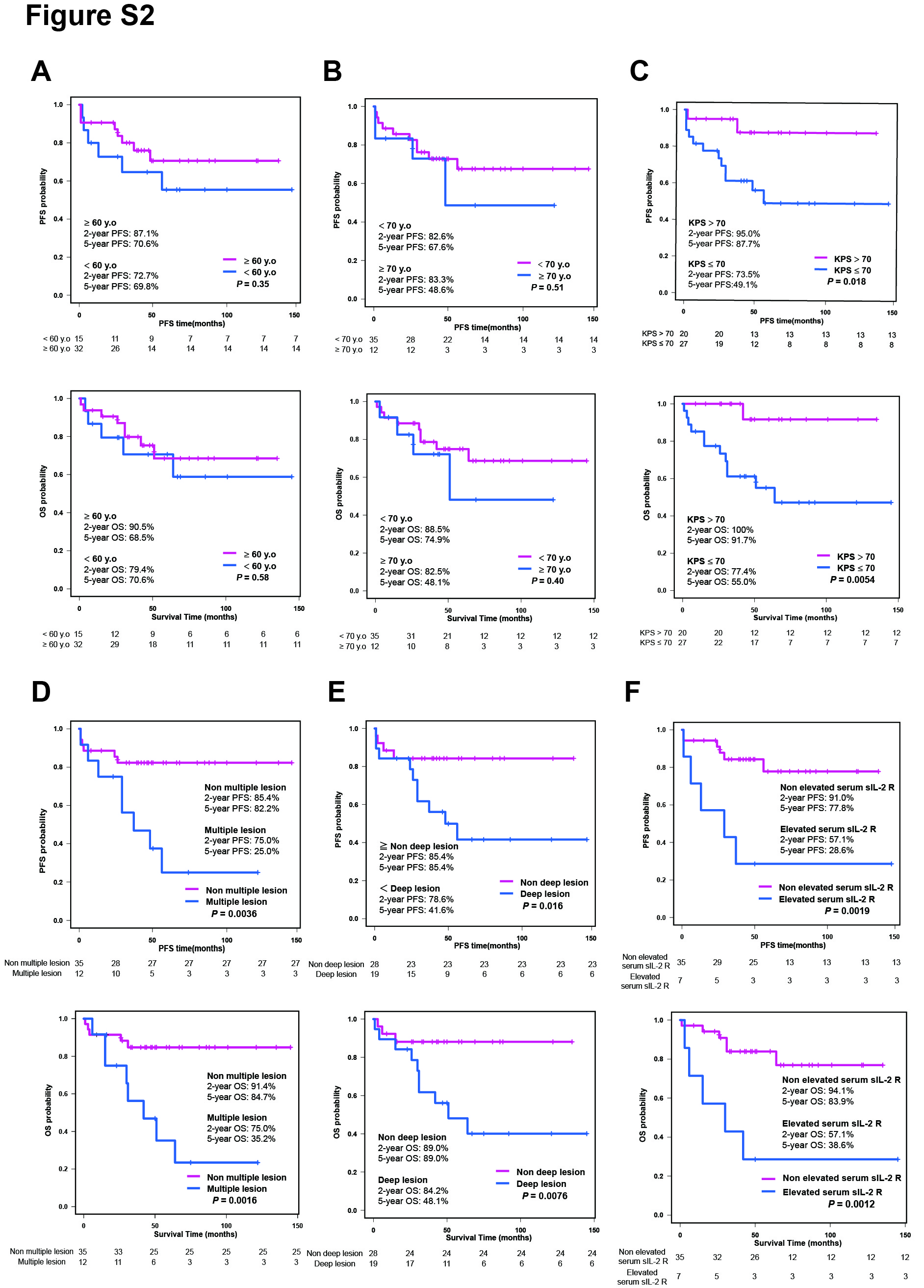

Supplement: Supplementary file 2 — Figure S2. [file CAM4-12-7116-s002.jpg]

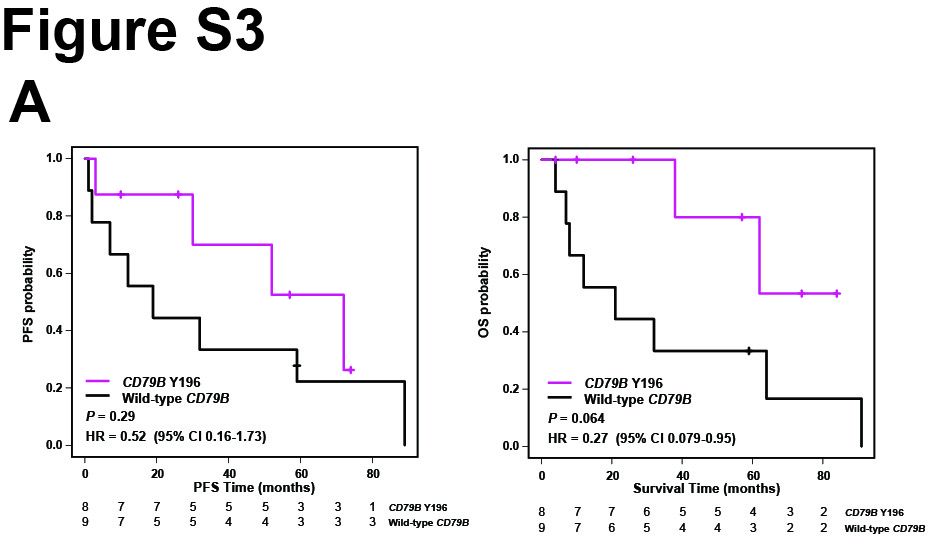

Supplement: Supplementary file 3 — Figure S3. [file CAM4-12-7116-s001.jpg]

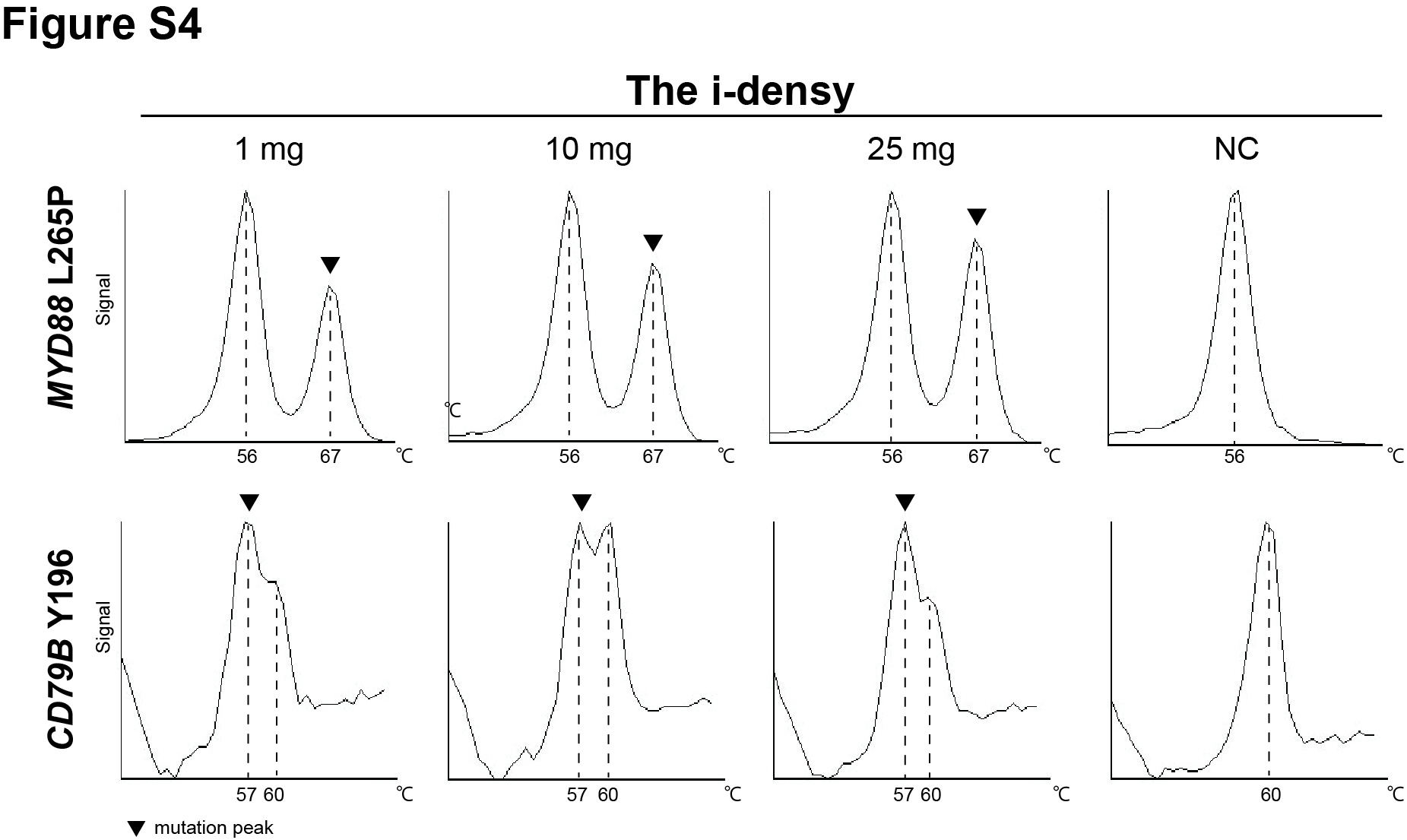

Supplement: Supplementary file 4 — Figure S4. [file CAM4-12-7116-s006.jpg]
